# Approaches for Selective Vaccinations in Cirrhotic Patients

**DOI:** 10.3390/vaccines11020460

**Published:** 2023-02-16

**Authors:** Giovanni Casella, Fabio Ingravalle, Adriana Ingravalle, Stefano Andreotti, Fulvio Bonetti, Claudio Monti, Rosanna Falbo, Maria Grazia Rumi

**Affiliations:** 1 General Practitioner Limbiate, 20812 Limbiate, Italy; 2 School of Specialization in Hygiene and Preventive Medicine, University of Rome Tor Vergata, 00133 Rome, Italy; 3 School of Specialization in Anesthesiology and Critical Care, University of Rome Sapienza, Policlinico Umberto I, 00133 Rome, Italy; 4 RSA “E.Bernardelli”, Clinica Polispecialistica San Carlo, 20037 Paderno Dugnano (Milan), Italy; 5 General Practitioner ATS Lecco-Monza Barlassina, 20825 Monza Brianza, Italy; 6 Clinical Pathology Department, Desio Hospital, 20832 Desio, Italy; 7 Hepatology Unit “San Giuseppe-Multimedica” Hospital, University of Milan, 20123 Milan, Italy

**Keywords:** cirrhotic patients, general practitioners, vaccination, chronic liver diseases, cirrhosis

## Abstract

Bacterial and viral infections are common in cirrhotic patients, and their occurrence is associated with the severity of liver disease. Bacterial infection may increase the probability of death by 3.75 times in patients with decompensated cirrhosis, with ranges of 30% at 1 month and 63% at 1 year after infection. We illustrate the indications and the modalities for vaccinating cirrhotic patients. This topic is important for general practitioners and specialists.

## 1. Introduction

Bacterial and viral infections/co-infections are frequent in cirrhotic patients [1,2]. The occurrence of infection is linked with the severity of liver disease [3]. Bacterial infection may increase the probability of death by 3.75-fold in patients with decompensated cirrhosis, with ranges of 30% at 1 month and 63% at 1 year after infection [1,2]. Bacterial and viral infections can deteriorate pre-existing liver damage and cause decompensation in cirrhotic patients [3,4]. Pneumococcal infections are more frequent and severe than other infections in cirrhotic patients with a higher rate of shock (13% versus 6%), bacteraemia (22% versus 13%), and death (14.4% versus 7.4%), compared to the general population [4]. Liver dysfunction determines an impaired immune function with a diminished synthetic production of complement proteins, pattern-recognition receptors, and acute phase reactants. This altered response to pathogens is combined with a paradoxical, chronic systemic inflammatory state, due to the immune cell upregulation of pathological cytokine production, specifically tumour necrosis factor-α (TNF-α), and surface activation markers [2].

## 2. Dimension of the Problem

The incidence of bacterial infections in cirrhotic patients during admission in hospitals is quite high. In several studies, 30% to 50% cirrhotics showed bacterial infections at admission or developed it during the period of hospitalization. Bacterial infections are frequently hospital-acquired infections. In fact, 15% to 35% of cirrhotic patients develop nosocomial infections, compared to 5% to 7% in the general hospital population. The need for frequent hospitalizations increases the risk of nosocomial infections in these patients [5]. Urinary tract infections (12% to 29%), spontaneous bacterial peritonitis (7% to 23%), respiratory tract infections (6% to 10%), and bacteraemia (4% to 9%) are the most frequent bacterial infectious complications [1]. All cirrhotic patients should be considered as immunocompromised patients, and the risk of infection increases as liver disease advances [6]. The dysfunction of the reticuloendothelial system, complement deficit for impaired liver synthesis, alteration of neutrophils, B and T lymphocytes, abnormal synthesis of immunoglobulins, and depressed macrophage function are the most important physio-pathological mechanisms of immunodepression in these patients [5]. Moreover, it is important to stress that some infections may be avoided by vaccine use [5]. Cirrhotic patients have both lower humoral and cellular immune response. Patients with decompensated cirrhosis even have a decreased response to vaccines and a loss of long-term immunogenicity, so early vaccination could be very important in patients with chronic liver disease, in order to improve the immune response [6]. Furthermore, cirrhotic patients do not have a higher risk of adverse effects related to vaccination, but they can be exposed to an increased risk during the use of live-attenuated vaccines [6]. Recommendations for vaccination in chronic liver disease have been well-defined by British [5] and American [7] associations, but guidelines are frequently discordant. Jacobs et al. [8] highlight that vaccination rates are lower in primary care than in specialist setting.

## 3. HAV Vaccination

Hepatitis A virus (HAV) vaccine is an inactivated virus vaccine, and it is well-tolerated in cirrhotic patients. However, the HAV vaccine triggers a reduced vaccination response in decompensated patients. The vaccination response rate, after two doses of HAV vaccine, is about 20–50%, compared to 99–100% response rate in immunocompetent subjects [9]. The British [5] and American [7] scientific societies recommend vaccination against HAV in every patient with chronic liver disease. Gutierrez Domingo et al. [10] found that, in 538 cirrhotic patients awaiting liver transplantation, 8.2% (44/538) were IgG-anti-HAV-negative and only 3 of these patients with IgG-anti- HAV (-) were over 60 years. The authors conclude that searching for HAV immunisation should always be recommended in patients under 45 years old during the evaluation before liver transplantation because HAV acute hepatitis may be fatal in these subjects [10]. Nowadays, the proportion of non-immune adults for HAV antigens is increasing, due to a decreasing incidence of hepatitis A [8]. Niriella et al. [11], in a cohort of 135 Sri Lankan cirrhotic patients, noted that none had received vaccination against hepatitis A, while 71 (67.6%) had been vaccinated against HBV. The majority of these (58%) were negative for IgG-anti-HAV, suggesting the importance of the HAV vaccine. Every cirrhotic patient should be routinely investigated for immunity against HAV, and vaccination should be offered to those found to be non-immune [11]. 

Italy is considered a country of low/intermediate incidence of HAV, with low endemicity in Northern and Central Italy and intermediate in the determined areas located in Southern Italy [12]. Considering the immune dysfunction in cirrhotic patients, Wigg et al. [13] studied immune responses of 73 patients with a standard dose (SD) of HAV vaccine (Havrix-Glaxo Smith Kline, Australia), intramuscular, 1440 mcg at 0 and 6 months, compared to high, accelerated dose (HAD) HAV (Havrix), 1440 mcg at 0, 1, and 2 months. HDA regimen showed a clinically significant 23% improved response rate (79.5%; 58 of 73 in SD arm, and 94.3%; 33 of 35 in HAD arm). In non-responding subjects, booster doses of Havrix of 1440 mcg in SD schedule and 720 mcg in HAD schedule were given, respectively, with successes of 66.7% in SD arm (8 of 12) and 100% (1 of 1) in the HAD arm [13] (Table 1).

## 4. Hepatitis HBV Vaccination

HBV is a well-tolerated vaccine, but its efficacy is impaired in cirrhotic patients [5]. The rate of HBV seroconversion after vaccination is about 40–70% in compensated cirrhotic patients and 30–50% in decompensated ones [8]. The British authority recommends HBV vaccination in all patients with HCV and alcoholic chronic liver disease. The United States authority recommends performing HBV vaccination at the earliest possible moment in patients awaiting liver transplantation [14]. HBV vaccines are available as a a single antigen formulation (HBsAg) or in combination with other vaccines, such as the HAV vaccine [15]. In the United States [15], the two recommended single antigen vaccines are Engerisx B (Glaxo Smith Kline Biologicals, Rixensart, Belgium) and Recombivax HB (Merck and Co., Inc, Whitehouse Station, Rahway, NJ, USA). Recombinant HBsAg and inactivated hepatitis A virus (Twinrix-Glaxo Smith Kline Biologicals, Rixensart, Belgium) may be used to vaccinate persons aged > 18 years [15]. It is important to recommend the control of blood antibodies post-vaccination and to propose additional injection in the case of inefficacy on the first immunisation schedule. Since immunosuppressed patients develop a less intense immune response alternative, strategies may include increasing the number of injections, doubling the dose of vaccine, or using adjuvant vaccines [5]. Attempts to immunise patients with liver cirrhosis have been proven relatively ineffective, and several strategies have already been used to improve the immune response in this group. The primary aim of this review is to examine, discuss, and summarise the immunogenicity of hepatitis B vaccination in patients with liver cirrhosis (Table 1). A MEDLINE search identified 11 studies (about 961 patients) on this field [16]. The dose of the vaccine and the schedule of the vaccination is variable. The response rates to HBV vaccination in cirrhotic patients range from 16% to 87%, regardless the doses of the HBV vaccine. In particular, it is important to underline that the patients who received a double vaccine dose revealed relatively better seroprotection rates (range: 26%–87%; mean response rate 53%), when compared to standard vaccination, in which seroprotection rates ranged from 16% to 79% (mean response rate 38%). The overall mean response rate to the HBV vaccination was 47%, suggesting that cirrhotic patients achieve lower seroprotection rates after the completion of a regular HBV vaccination series. However, there is a great need for additional studies to analyse the immune response, in relation to poor vaccination response, to study new strategies to improve immunogenicity, and to better understand the immune mechanism underlying the differences in the response rates to HBV vaccination [8]. Wigg et al. [13] studied 97 cirrhotic patients with two different vaccination schemes. A standard dose (SD) regimen (Engerix B Glaxo Smith Kline), 20 mcg at 0, 1, and 6 months, with a 40 mcg booster of Engerix B if a non-responder, and a high, accelerated dose (HAD) at the dosage of 40 mcg at times 0, 1, and 2 months with a booster of 40 mcg if a non-responder. The initial response was 51.5% (50 of 97) in the SD arm and 45.1% (23 of 51) in the HAD arm, but a booster dose induced seroconversion in 28.6% (12 of 42) in the SD arm and 52.6% (10 of 19) in the HAD arm [13], with a final response of 67.4% (62 of 92) in the SD arm and 78.6% (33 of 42) in the HAD arm [13] The HAD boosting regimen may be considered highly useful [13]. On multivariate analysis, low levels of albumin were associated with immune non-response [13]. Yanny et al. [17], in their review, analysed four studies concerning HBV vaccine non-responder cirrhotic patients, for a total of 83 patients. They found that a new complete HBV vaccination schedule with three intramuscular doses of 10 or 20 mcg permitted a seroconversion rate of 82.7% in hypoalbuminemic patients, while a single-dose booster increased seroconversion to 38.2%; a higher dose of 40 or 60 mcg did not increase seroconversion. De Artaza et al. [18] studied 57 cirrhotic patients who received three doses of a 20 μg HBV vaccine; they showed that 47.3% of the patients achieved seroprotection. Moreover, they found that the efficacy increased to 68.4% if a fourth double dose was administered. In another study, Aziz et al. [19] analysed the response to three doses of 80 μg vaccine in patients who had previously failed with a monthly three-dose 40 μg IM vaccine; a total of 52% of the cirrhotic patients developed immunity. 

Hepatitis B virus and hepatitis C virus (HCV) co-infection increases the severity of hepatitis, determining an additional risk for developing liver cirrhosis and hepatocellular carcinoma. Whether chronic HCV infection decreases antibody response to hepatitis B vaccination is still controversial [20]. Liu et al. [20] evaluated the influence of HCV infection on antibody response to hepatitis B vaccination by a systematic review of published works with a meta-analysis of clinical trials. They identified 11 studies involving 704 patients with HCV and 812 controls, revealing a significant decrease in antibody seroconversion rates among patients with HCV versus healthy controls (pooled odds ratio  =  0.17 (95% confidence interval, 0.11–0.28)). However, Liu et al. [20] concluded that both cirrhotic and non-cirrhotic patients with hepatitis C infection have a statistically significant lower rate of seroconversion, in comparison to the healthy controls. Chronic HCV infection can decrease the immune response to a standard schedule of hepatitis B vaccination. Further studies are needed to investigate the optimum vaccination schedule for patients with chronic HCV infection [20]. Recently, a new hepatitis B vaccine, licensed in USA as the CPg-adjuvanted hepatitis B vaccine (HEPLISAV-BR) [21], offers a higher seroprotection in liver disease patients, compared to the traditional three-dose vaccine (Engerix B) (63% vs. 45% *p* = 0.03). The presence of cirrhosis, chronic obstructive pulmonary disease, or renal failure may determine a lower immunological response [21].

## 5. Flu Vaccination

Influenza virus infection can increase the risk of decompensation in liver cirrhotic patients [22]. In fact, influenza is more severe and it is linked with a higher risk of organ failure, which can be responsible of the patient’s death [22]. However, flu vaccination is still important because of the increased morbidity and mortality in cirrhotic patients with the flu [23]. Sayyad et al. [24] performed a study to assess the immunogenicity of flu vaccination in patients with both cirrhosis and inactive hepatitis B virus; the results were similar to the health population, confirming the indication for vaccination in these patients. Cheong et al. [25] enrolled 93 subjects, divided into 3 groups: cirrhotic (*N* = 28), inactive carriers of hepatitis B (*N* = 31), and normal subjects (*N* = 34). The flu vaccine (Influvac^®^) was administered in all the participants. To test the humoral immunogenicity by the hemagglutination inhibition (HI) test, a simple blood sample was taken before the vaccination and 4 weeks after vaccination. Four weeks after vaccination, seroconversion rates ranged between 71.4% and 100% in Group 1, 70.6% and 94.1% in Group 2, and 58.1% and 80.7% in Group 3. No significant differences were seen in the rates of seroconversion and antibody geometric nean titers (GMTs) against the flu A (H1N1 and H3N2) vaccine components in the three groups (*p* < 0.05). The rates of seroconversion and antibody GMTs against the flu vaccine components were significantly higher in cirrhotic and inactive carriers of hepatitis B than in healthy subjects (*p* < 0.005). No significant differences (*p* < 0.05) in the rates of seroprotection were observed within the three groups. Antibody GMTs against all three strains of the flu vaccine increased significantly (*p* < 0.001) after vaccination in the three groups. Hence, their study showed a similar humoral immune response in both the cirrhotic patients and healthy patients [25]. Therefore, the flu vaccine is effective in cirrhotic patients and inactive carriers of hepatitis B, as well as healthy individuals. It means that vaccination should be considered in such patients, in order to reduce the morbidity and mortality of the flu [24,25] (Table 1). The flu vaccine may be considered safe in patients with chronic liver disease, even if a few side effects, as “Guillan-Barre’ Syndrome”, have been reported in the general population [26]. The safety of the adjuvanted influenza vaccine had been confirmed in patients with decompensated cirrhosis [27] and in chronic liver diseases [28].

## 6. Pneumococcal Vaccination

The clinical course of pneumococcal infection in cirrhotic patients is more serious than in the general population. Kyaw et al. [22], in a retrospective study of 2765 cases with pneumococcal infection, have found that the risk of invasive infection is higher in chronic patients, with a relative risk (RR) of 7.4 (95% CI3.2–16.9). Cirrhotic patients show an increased risk of bacteraemia during pneumococcal infection, with an RR of 11.4 (95% CI5.9–21.9) [6]. The risk of death for pneumococcal infection is higher in cirrhotic patients, without other factors of immunosuppression, compared to the general population [29]. Gransden et al. [30], in their retrospective study, have also observed that liver cirrhosis can be a factor of poor prognosis during pneumoniae bacteraemia, confirming the opportunity and the importance of pneumococcal vaccination in cirrhotic patients [5]. The anti-pneumococcal vaccine has good safety in this type of subject [5]. However, McCashland et al. [31] noted that cirrhotic patients have a lower immunogenicity and a faster decline of humoral response (IgG). The patients should be vaccinated once with pneumococcal conjugate vaccine (PCV13), followed by pneumococcal polysaccharide vaccine (PPSV23), generally 1 year after [32]; a second dose of PPSV23 could be suggested 5 years after the first dose, with a third dose after 65 years of age [32]. If the patient receives PPSV23 as the initial vaccine (before), PCV 13 must be administered always 1 year after [33]. However, following the introduction of 15-valent PCV (PCV 15) and 20-valent PCV (PCV 20), PCV 13 is no longer recommended in patients aged 19–64 years with chronic liver disease. Moreover, when PCV 15 is administrated, after 1 year, a following dose of PPSV23 should be administered [34] (Table 1). More studies are needed. 

## 7. Varicella Zoster Virus (VZV) Vaccination

VZV infection has a characteristic bimodal age distribution. The first peak of incidence generally occurs between 1 and 9 years of age, with a “classic” active varicella (chickenpox) infection. After this “classic” infection, the virus becomes “dormant” within the dorsal root ganglia and may reactivate during an impaired immunological response, producing the pathognomonic dermatological manifestations of VZV. In 6–30% of immunocompromised patients, the reactivated virus can cause disseminated cutaneous VZV infection. This infection is characterized by the presence of more than 20 vesicular lesions, and rashes may occur widely on the patient’s body [35]. These cutaneous manifestations should be considered an “alert flag”, since they can anticipate a life-threatening condition, such as multi-organ involvement, including encephalitis, meningitis, pneumonitis, and myocarditis. The recent Zostavax administration, in the case of the cutaneous eruption, has shown interesting results [36]. Cheetham et al. [36], in a study of over 14,000 patients taking immunosuppressive medications and, at the same time, receiving the VZV vaccine, showed no link to disseminated VZV outbreaks. However, several reports describe disseminated VZV related to the Oka VZV strain [37]. The outbreaks usually occur within 42 days of vaccination. It is difficult to ascertain whether the outbreaks were due to the vaccine or reactivation of the native virus, but the timing of events certainly raises that clinical question. Cirrhotic patients have an alteration in cell mediated immunity, particularly in end-stage liver disease (ESLD), and this may explain a particular caution when using this type of vaccine in these subjects. Patients without history of prior varicella infection), especially in children with cirrhosis, should be screened for antibodies against Varicella-Zoster Virus (VZV). The recombinant HZ (subunit) vaccine (RZV; Shingrix, GlaxoSmithKline) is constituted by glycoprotein E, associated with a novel adjuvant permitting the amplification of the immune response [38]. This vaccine is administered as a series of two doses, separated by 2 to 6 months [38]. RZV is highly immunogenic with 97% efficacy in individuals aged 50 years and older and 89% efficacy in individuals aged 70 years and older in a clinical trial [39] (Table 1). L’Huillier et al. [40] recommended the use of recombinant varicella vaccine in patients who are candidates for a liver transplant before the transplantation. The live attenuated zoster vaccine (ZVL) licensed in 2006 is no longer available in the United States, as of November 2020 [41]. Shingrix is the alternative (not may actually be the preferred vaccine) for the prevention of shingles; only future trials shall evaluate whether Shingrix might be an alternative for primary vaccination in the future. 

## 8. COVID-19 Vaccination

The coronavirus disease 2019 (COVID-19) pandemic has changed the life of the entire world’s population [42]. At the end of 2020, the vaccination for COVID 19 started and it has reduced the morbidity and the mortality in the general population [42]. The available vaccines are RNA-based vaccines and named BNT162b2 mRNA (BioNtech and Pfizer) and MRNA-1273 (Moderna) [42]. The BNT162b2 mRNA (BioNtech and Pfizer) has been demonstrated to be safe and effective; the vaccine was intramuscularly administered in two doses at an interval of 21 days (day 0 and day 21). MRNA-1273 (Moderna) is safe and effective as a BNT162b2 mRNA (BioNtech and Pfizer), and it is intramuscularly administered at an interval of 28 days (day 0 and day 28) (Table 1). 

Both vaccines show a good safety profile and protect against the SARS-CoV-2 infection in 95% of cases (95% credible interval of 89.3–96.8%). The ChAdOx1 nCOV-19 vaccine and the AZD1222 vaccine, also known as Covishield (Astra Zeneca and University of Oxford), is based on the chimpanzee adenovirus. This type of vaccine works as a vector, in fact it shows the spike protein of the virus. The overall vaccine efficacy is 79.4%. In March 2021, the EMA’s safety committee performed a preliminary review concerning the safety of AstraZeneca because some reports had shown the formation of blood clots in subjects vaccinated with the ChAdOx1 nCOVID-19 vaccine. The EMA’s safety committee concluded that the utility derived from the vaccination exceeds the reported side effects and that the vaccine is not recommended for patients with thrombocytopenia. The vaccines approved by the European medical agency (EMA) have been demonstrated to be safe for the general population [43]. 

The Johnson & Johnson/Janssen vaccine uses a replication-incompetent adenoviral vector that contains the DNA encoding for the spike glycoprotein [44]. The Advisory Committees on Immunization Practices (ACIP) recommends the Pfizer-BioNTech or Moderna mRNA COVID-19 vaccines over the Johnson & Johnson/Janssen COVID-19 vaccine for primary and booster vaccinations [45]. Patients with cirrhosis, especially with decompensated cirrhosis, are reported to have an increased risk of SARS-CoV-2 infection and worse outcomes [46,47]. The higher risk to contract the infection is due to their compromised immune system; acute decompensation in cirrhosis was observed in 20–46% of the cirrhotic patients with COVID-19 disease [42]. Several studies from Asia, North America, and Europe confirmed that cirrhotic patients have five-fold higher risk to develop severe COVID 19 infection with increased risk of mortality (three-fold higher risk). Age, Child-Pugh Class B or C, MELD score and comorbidities are all factors associated with an increased mortality in cirrhotic patients [48]. The cirrhotic patients awaiting liver transplantation should be early vaccinated for COVID 19 infection, due to high-risk mortality in the pre-transplant phase [42]. 

A prospective, multi-centre, open-label study enrolled 581 Chinese patients with chronic liver disease (CLD) [49]. The patients enrolled received two doses of inactivated whole-virion SARS-CoV-2 vaccines, and its safety and immunogenicity were evaluated. The authors selected (performed) three groups based on different stages (statuses) of cirrhosis and compensation. The time interval between the first and second doses was 3 to 8 weeks; after 14 days of the second dose, a serum sample was taken, and it was tested for SARS-CoV-2 neutralising antibodies. Their study [49] suggested that the whole schedule (two doses) of inactivated whole-virion SARS-CoV-2 vaccination is relatively safe in patients with CLD; however, these types of patients have lower immunologic response to SARS-CoV-2 vaccines than the healthy population. These results were in accordance with those found in the literature, where patients with liver disease had shown lower response to other vaccines, such as HBV vaccines [16,48,50]. A decreased response might be due to the impairment of the immune system and to the male gender. Therefore, they suggest a boosting dose of SARS-CoV-2 vaccination in patients with CLD to get a better response [49,50].

It is important to stress the importance of a vaccine priority in patients with medical conditions that increase the risk of severe illness from COVID-19 as patients with chronic liver disease, such as NAFLD, liver cirrhosis, biliary atresia, who had the characteristics of priority [51,52]. Especially, NAFLD with concomitant SARS-CoV-2 infection is linked to worse outcomes [53,54]. 

## 9. Tetanus, Diptheria, and Pertussis (Tdap) Vaccine

The data about the efficacy of these vaccines in patients with chronic liver diseases (CLD) are limited [55]. ACIP recommend one dose of Tdap in CLD patients, followed by Tdap booster every ten years [56] (Table 1). 

## 10. Measles, Rubeola, and Mumps (MMR) Vaccine

Measles, rubeola, and mumps are viruses that can cause severe disease, in particular in immunocompromised patients [41]. MMR vaccination is strongly recommended in all general population worldwide. This indication also applies to patients with liver cirrhosis or liver disease (Table 1), although the available data are limited [55]. If IgG MMRs are undetectable, a booster dose of the vaccine should be given in patients with liver disease. In the case that liver transplantation is planned, the ACIP recommendations suggest anticipating the MMR vaccination. In children, vaccination 1 year after transplantation is possible in the case of low levels of immune suppression [57].

## 11. Prevalence of Vaccination Rate in Cirrhotic Patients

Arguedas et al. [9] reported that immunisation coverage in cirrhotic patients awaiting liver transplantation was 26% for HBV and HAV infection, 55% for flu infection, and 34% for pneumococcal infection. Denis et al. [58], in a retrospective study about hepatitis HCV patients, found HAV vaccination only in 27% of non-immunized subjects. Mazzola et al. [59], in their study on 222 patients awaiting liver transplantation, noted that only 26 (14.6%) were vaccinated for HAV hepatitis, 63 (36%) for HBV hepatitis, and 111 (62.3%) for tetanus. Additionally, of the 222 patients [59], only 24 cirrhotic patients have vaccination records. Currently, vaccination against HAV and HBV is universally suggested in patients with chronic diseases, but in Mazzola’s study [59], 25.8% of cirrhotic patients did not show blood HAV antibodies, and in a population with only 11.6% of patients with active HBV infection, more than 50% were not protected for HBV infection. The data in the scientific literature suggest that not all general practitioners and specialists are well-informed about vaccination policies. Vaccination in cirrhotic patients should be strongly recommended to increase the vaccination coverage rate [60]. The behaviour of general practitioners (GPs) and specialists is different, regarding the use of vaccination: GPs perform more vaccination against influenza and pneumococcal infection, while specialists more often use vaccination for HAV and HBV [8]. The progression of chronic liver disease to cirrhosis involves both innate and adaptive immune system dysfunction, resulting in an increased risk of infectious complications. Vaccinations against pneumococcus, HAV, and HBV are well-tolerated and effective in disease prevention and in morbidity and mortality reduction. Prior studies assessing vaccination rates in cirrhotic patients have specific limitations, and no previous study has provided a comprehensive evaluation of vaccination rates in patients with cirrhosis in the United States. Waghray et al. [61] assessed a study regarding vaccination rates for pneumococcus, HAV, and HBV in patients with cirrhosis and found that only 59.7% of patients with cirrhosis had received at least one vaccination. Vaccination rates within the same or following year of cirrhosis diagnosis were 19.9%, 7.7%, and 11.0% against pneumococcus, HAV, and HBV, respectively. This study revealed significant increases in vaccination rates for pneumococcus in all patients with cirrhosis and within subgroups based on age, gender, and the presence of concomitant diabetes. Although the vaccination rates in patients with cirrhosis remain suboptimal, the use of electronic medical record (EMR) reminders may improve the communication between healthcare professionals and public health officers, in order to increase the awareness of vaccinations, in order to reduce morbidity, mortality, and likely, healthcare-related costs of vaccine-preventable diseases in cirrhotic patients [61]. There are several solutions in aiding to spread the use of vaccination: physician education, scheduled reminders, and recall systems [62]. Recently, an electronic health system has been used to improve vaccination coverage by an automatic reminder for chronic liver disease patients, which made it possible to improve the vaccination coverage rate [63].

## 12. Conclusions

Vaccination is a simple way to prevent a great number of infections in cirrhotic patients. HAV and HBV vaccination should be strongly recommended in all non-immunized patients with liver cirrhosis, as well as flu and pneumococcal vaccines. A vaccination recall should also be suggested for diphtheria, tetanus, and poliomyelitis. Live attenuated vaccines are not contraindicated in cirrhotic patients, but the risks and benefits should always be considered. Lastly, the recent SARS-CoV-2 vaccine should be considered in cirrhotic patients, in order to avoid a severe COVID-19 disease. The future challenge is to spread the recommendation about vaccination in cirrhotic patients among healthcare professionals. An increased use of vaccinations will allow us to avoid severe infectious disease, with a subsequent decrease of morbidity and mortality. Cirrhotic patients can have more advantages and less complications if they are well-instructed about the importance of vaccinations.

## Figures and Tables

**Table 1:** Vaccination Schedule.

Vaccinations	Schedule
HAV Hepatitis	2 doses at 1–6 months
HBV Hepatitis	3 doses at 0–1–6 months with old vaccines and 2 doses at 0–1 months with new vaccine HEPB-CpG
Flu	Annual with inactivated virus
Pneumococcus	PCV 20 or PCV15 followed by PPSV23 1 year after but 2 months after in particular immunocompromised patients
Meningococcus	2–3 doses regimen suggested, particularly in endemic areas, patients with complement deficiencies, and hypo-asplenism
Herpes Zoster	2 doses at 0–2 months in patients aged over 50 years of recombinant Zoster vaccine
COVID-19 BNT162b2 mRNA (BioNtech and Pfizer) COVID-19 MRNA-1273 (Moderna)	At time 0 and after 21 days At time 0 and after 28 days
Tetanus, Diphteriae, and Pertussis	Recall and revaccinate every 10 years
Measles, Rubeola, and Mumps	Every measle negative subject should be vaccinated if there is no contraindication to a live vaccine

## Data Availability

Not applicable.
